# Antidepressant mechanism of ketamine: perspective from preclinical studies

**DOI:** 10.3389/fnins.2015.00249

**Published:** 2015-07-21

**Authors:** Lisa Scheuing, Chi-Tso Chiu, Hsiao-Mei Liao, De-Maw Chuang

**Affiliations:** Molecular Neurobiology Section, National Institute of Mental Health, National Institutes of HealthBethesda, MD, USA

**Keywords:** ketamine, NMDA antagonist, major depressive disorder, lithium, GSK-3 inhibitor, mTOR, therapeutic potential

## Abstract

A debilitating mental disorder, major depressive disorder is a leading cause of global disease burden. Existing antidepressant drugs are not adequate for the majority of depressed patients, and large clinical studies have demonstrated their limited efficacy and slow response onset. Growing evidence of low-dose ketamine's rapid and potent antidepressant effects offers strong potential for future antidepressant agents. However, ketamine has considerable drawbacks such as its abuse potential, psychomimetic effects, and increased oxidative stress in the brain, thus limiting its widespread clinical use. To develop superior antidepressant drugs, it is crucial to better understand ketamine's antidepressant mechanism of action. Recent preclinical studies indicate that ketamine's antidepressant mechanism involves mammalian target of rapamycin pathway activation and subsequent synaptogenesis in the prefrontal cortex, as well as glycogen synthase kinase-3 beta (GSK-3β) inactivation. Adjunct GSK-3β inhibitors, such as lithium, can enhance ketamine's efficacy by augmenting and prolonging its antidepressant effects. Given the potential for depressive relapses, lithium in addition to ketamine is a promising solution for this clinical issue.

## Introduction

A devastating mental disorder, major depressive disorder (MDD) is a leading global disease burden and is currently the eleventh highest contributor to global disability-adjusted life years (Murray et al., 2012). In the United States alone, MDD remains the second highest contributor to years lived with disability, and recently rose to the fifth leading contributor to disability-adjusted life years according to epidemiological studies from 1990 to 2010 (Collaborators, 2013). The mental health community is largely in agreement that current antidepressant drugs are not adequate due to the long treatment time course required to reach full efficacy (weeks to months), and their limited response in treatment-resistant patients (Insel and Wang, 2009). Many depressed patients, especially those who are at risk for suicide, require an effective, fast-acting antidepressant. Ketamine is a non-competitive glutamate *N*-methyl-D-aspartate (NMDA) receptor antagonist, and has been widely used as an anesthetic agent (Sanacora and Schatzberg, 2015). During the past 15 years, numerous clinical studies have provided strong evidence that a single sub-anesthetic dose of ketamine rapidly and robustly alleviates depressive symptoms in MDD patients. Additionally, animal models of depression have allowed researchers to unravel ketamine's unique antidepressant mechanism of action. In this review we will discuss the clinical usage of ketamine, highlight seminal papers that elucidate ketamine's antidepressant mechanism, and propose future directions for this promising developing field.

## Clinical usage of ketamine as an antidepressant

It is now widely accepted that ketamine is an effective and fast-acting antidepressant, proven to be beneficial for a variety of depressed patients. The first clinical study to assess the antidepressant effects of ketamine at a dose of 0.5 mg/kg, given intravenously over 40 min, significantly reduced depressive symptoms in MDD patients within 24 h, compared with saline placebo (Berman et al., 2000). However, ketamine-treated patients experienced dissociative side effects during the first 2 h post-infusion. A subsequent trial demonstrated ketamine's effectiveness in treatment-resistant MDD, which is defined as inadequate response to more than two antidepressants (Zarate et al., 2006). However, a single ketamine infusion was not long-lasting, with 35% of patients maintaining a significant antidepressant response for up to 1 week. While the majority of clinical studies reported the effects of a single ketamine administration, repeated ketamine therapy of six infusions over 12 days was safe and well tolerated in treatment-resistant MDD patients (Aan Het Rot et al., 2010). Further evidence of successful repeated ketamine therapy reported an overall response rate of 70.8% for treatment-resistant patients, with a median depressive relapse time of 18 days (Murrough et al., 2013b). Compared with an average of 7 days until non-significant response or depressive relapse, 18 days until depressive relapse demonstrates strong improvement. To compensate for the initial dissociative symptoms of ketamine, a large double-blind study used a psychoactive placebo, midazolam, to compare antidepressant effectiveness and described response rates of 64% for ketamine but only 28% for midazolam (Murrough et al., 2013a). While there is no perfect placebo for ketamine, the use of midazolam is a better substitute than saline because it blinds patients to treatment.

In sharp contrast to the slow onset of existing antidepressants, ketamine's fast-acting effects provide relief for MDD patients at risk for suicide. For example, ketamine infusions rapidly decreased explicit suicidal ideation in MDD patients within 24 h (Price et al., 2009), even when compared to midazolam (Price et al., 2014). In addition to MDD, ketamine is an effective antidepressant for bipolar-depressed patients as demonstrated by double-blind, saline placebo-controlled studies, in which patients had response rates of 70% or greater to ketamine (Diazgranados et al., 2010; Zarate et al., 2012b). Ketamine has also shown promise to decrease symptoms in both obsessive-compulsive disorder (Rodriguez et al., 2013) and post-traumatic stress disorder (Feder et al., 2014) patients. However, further double-blind trials with appropriate placebos are required to assess ketamine's long-term effects, in addition to its potential to treat psychiatric disorders besides MDD.

## Ketamine's antidepressant mechanism of action

It was first reported that MK-801, a non-competitive NMDA receptor antagonist, exhibited antidepressant-like actions in mice (Trullas and Skolnick, 1990). A later preclinical study demonstrated that ketamine injected male rats showed significantly less immobility during the forced swim test (FST) compared with saline controls, and inferred that the animals exhibited less behavioral despair than controls (Yilmaz et al., 2002). In addition, ketamine-injected male rats had reduced immobility time in the FST and had increased hippocampal brain-derived neurotrophic factor (BDNF) protein levels in comparison to untreated rats, suggesting involvement of the tropomyosin receptor kinase B (TrkB) signaling pathway (Garcia et al., 2008). Mice that underwent the learned helplessness paradigm were also responsive to ketamine treatment as demonstrated by decreased immobility times in the FST, and pre-treatment with an α-amino-3-hydroxy-5-methylisoxazole-4-propionic acid (AMPA) receptor antagonist NBQX attenuated the antidepressant-like behavior induced by ketamine, suggesting that pre-synaptic NMDA receptor blockade and post-synaptic AMPA receptor activation and upregulation is necessary for ketamine's antidepressant effects (Maeng et al., 2008).

In a pivotal study, researchers demonstrated that ketamine rapidly and transiently activated the mammalian target of rapamycin (mTOR) signaling pathway in the medial prefrontal cortex (mPFC) of male rats (Li et al., 2010). Evidence of mTOR activation was supported by increased phosphorylation of upstream proteins including extracellular signal-regulated kinase (ERK; forms 1 and 2) and protein kinase B (PKB/Akt), and downstream proteins p70 ribosomal protein S6 kinase (p70S6K) and translation repressor protein 4E-binding protein 1 (4E-BP1). The phosphorylation increases for the respective active protein forms were dose-dependent, with peak activation occurring at low ketamine doses (5–10 mg/kg), and were transient, lasting up to 2 h. Stimulation of p70S6K increases the synthesis of ribosomal unit S6, and overall increases protein translation (Fenton and Gout, 2011), whereas the hyper-phosphorylation of 4E-BP1 dis-inhibits protein translation by decreasing eukaryotic initiation factor 4E (eIF4E) activity, allowing for the recruitment of translation machinery to mRNA (Gingras et al., 2001). Ketamine-induced mTOR activation also significantly increased mRNA and protein levels of activity-regulated cytoskeletal-associated protein (Arc), synapsin I, postsynaptic density protein 95 (PSD95), and GluR1 (an AMPA receptor subunit), all proteins critically involved in the formation, maturation and function of new spine synapses (Li et al., 2010). In support of behavioral despair model findings, ketamine treatment reversed behavioral and synaptic spine deficits caused by chronic unpredictable stress, which induced anhedonia- and anxiety-like behaviors; ketamine alleviated decreased synaptic density protein levels of synapsin I, PSD95, and GluR1within the rat mPFC (Li et al., 2011).

Preclinical findings of significantly lower synaptic spine density proteins in animal depression models have been reinforced by post-mortem studies of MDD subjects who had reduced levels of PSD95 (Feyissa et al., 2009), as well as significantly lower protein levels of mTOR and p70S6K (Jernigan et al., 2011), in the PFC compared to sex- and age-matched controls. Unlike other widely prescribed antidepressants such as imipramine or fluoxetine, activation of the mTOR pathway and its associated increase in synaptic spine density within the mPFC is one unique mechanism underlying ketamine's antidepressant-like effects (Park et al., 2014). It is important to note that there are two complexes associated with mTOR, complex 1 (mTORC1) which requires regulatory-associated protein of mTOR (Kim et al., 2002), and complex 2 (mTORC2) which contains rapamycin-independent companion of mTOR (Sarbassov et al., 2004). While mTORC2 could potentially regulate ketamine's antidepressant effects, the fact that rapamycin pre-treatment prevents the antidepressant actions of ketamine, given that rapamycin has a greater affinity for mTORC1 (Sarbassov et al., 2005), strongly suggests that mTORC1 is the primary target (Li et al., 2010, 2011). Future, studies should use specific mTOR complex inhibitors to determine which mTOR complex is necessary for the different molecular events of ketamine's antidepressant mechanism.

Besides mTOR activation, another important process required for ketamine's antidepressant effects is the production and release of BDNF, a neurotrophic factor essential for neuron development, survival, and synaptic plasticity (Autry and Monteggia, 2012). One mechanism explains that NMDA receptor blockade leads to downstream inhibition of eukaryotic elongation factor 2 kinase (eEF2K) by p70S6K, thus decreasing the phosphorylation of eEF2K's target protein, eEF2, and overall de-suppressing protein translation machinery, resulting in enhanced BDNF expression within the hippocampus (Autry et al., 2011). Unlike the uniqueness of the rapid and transient mTOR pathway activation, enhanced BDNF expression is a common event for both ketamine and classical antidepressants (Monteggia and Zarate, 2015). Increased BDNF production and release is critical for ketamine's rapid mechanism of action, demonstrated by the lack of antidepressant-like response to ketamine in BDNF-conditional knockout mutant mice (Autry et al., 2011), or in mice infused with a neutralizing BDNF antibody in the extracellular space of the mPFC (Lepack et al., 2014).

Another mechanism proposes that ketamine causes increased BDNF signaling via post-synaptic AMPA receptor stimulation, depolarizing the cell, activating L-type voltage-dependent calcium channels, thus allowing for calcium influx and resultant activity-dependent exocytosis of BDNF (Lepack et al., 2014). BDNF then activates the TrkB signaling pathway, causing phosphorylation and activation of downstream effector proteins ERK1/2 and Akt through the MEK and PI3K signaling pathways, respectively. BDNF upregulation due to ketamine administration also leads to acute changes in synaptic plasticity (increased surface expression of AMPA receptors) within the hippocampus (Nosyreva et al., 2013; see Kavalali and Monteggia, 2012 for details about ketamine and synaptic plasticity).

Further, support of BDNF's involvement stems from the decreased antidepressant-like effectiveness of ketamine in genetically engineered mice carrying at least one allele with methionine substituted for valine of the BDNF Val66Met polymorphism (Liu et al., 2012), which has also been confirmed in a small sample of human MDD patients (Laje et al., 2012). Having two methionine alleles (met/met) of the Val66Met polymorphism reduces BDNF production and release, and cognitively is correlated with poorer episodic memory (Egan et al., 2003). It has been suggested that because 25% of the general population is estimated to have the Val66Met polymorphism (Petryshen et al., 2010); this may potentially explain why approximately one third of MDD patients are non-responders to ketamine. However, it is likely that a more complex combination of factors is why some MDD patients are unresponsive to ketamine treatment.

**Figure 1:**
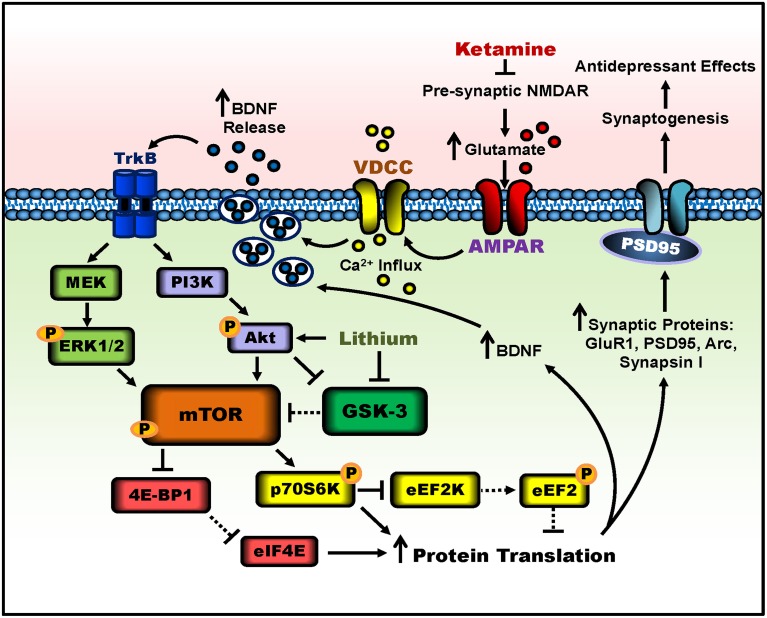
**Putative signaling pathways involved in ketamine's antidepressant effects and the potentiation by lithium**. Ketamine blocks pre-synaptic NMDAR signaling, resulting in increased glutamate release. Enhanced glutamate signaling activates post-synaptic AMPA receptors, and the resultant cell depolarization stimulates voltage-dependent calcium channels (VDCC), leading to calcium influx and BDNF exocytosis. BDNF release activates TrkB receptors and downstream signaling pathways, PI3K-Akt and MEK-Erk1/2. Both pathways activate mTOR complex 1 through phosphorylation. The activity of mTOR can be potentiated by lithium through Akt activation and GSK-3 inhibition. mTOR then phosphorylates and activates p70S6K, which inhibits eEF2K, halting the phosphorylation of eEF2, effectively inhibiting eEF2. In parallel, mTOR hyperphosphorylates 4E-BP1, reducing its interaction with eIF4E. Together, decreased eEF2 phosphorylation and the release of eIF4E from 4E-BP1 disinhibit protein translation, producing more synaptic proteins such as GluR1, PSD95, Arc, and synapsin I, as well as BDNF. This facilitates increased dendritic spine density and synaptogenesis in the prefrontal cortex and hippocampus, and leads to antidepressant-like behavior in rodents. Lines with arrows represent stimulatory connections; lines with flattened ends represent inhibitory connections. Dashed lines represent pathways with reduced activity as a result of ketamine or lithium treatment.

Another significant protein called glycogen synthase kinase-3 (GSK-3), a master switch serine-threonine kinase implicated in psychiatric disorders such as MDD and bipolar disorder (Beurel et al., 2015), undergoes necessary inhibition for ketamine's rapid antidepressant effects. Researchers used a knock-in mouse model rendering both the alpha and beta isoforms of GSK-3 maximally active, by eliminating the inhibitory phosphorylation sites of serine-21 and -9 for GSK-3α and GSK-3β, respectively (Beurel et al., 2011). When GSK-3 could not be inhibited, the antidepressant-like effects of ketamine were not observed in the learned helplessness paradigm. Thus, GSK-3 inhibition is also necessary for ketamine's rapid antidepressant-like effects. Building upon the requirement of decreased GSK-3 activity, two separate preclinical studies have demonstrated that lithium, a GSK-3 inhibitor, combined with low-dose ketamine potentiated and prolonged the molecular and behavioral effects of ketamine (Liu et al., 2013; Chiu et al., 2014), see Figure 1 for a schematic. These two studies will be discussed in depth in the following section.

## Issues with ketamine treatment and possible solutions

Ketamine infusions have proven to be beneficial for patients with treatment-resistant depression, however, ketamine also temporarily causes dissociative symptoms, has risk for abuse, and has been shown preclinically to increase oxidative stress in the rat brain (Zuo et al., 2007). In addition, it is evident from both clinical and preclinical studies that the antidepressant effects induced by a single ketamine administration usually last 1 week. Therefore, although ketamine rapidly reduces depressive symptoms, repeated infusions are necessary to maintain its effects. However, repeated ketamine administrations can cause a variety of side effects in humans such as cognitive impairments and psychomimetic symptoms (Krystal et al., 2005). Moreover, administration of sub-anesthetic doses of ketamine in animals has been used as a pharmacological model of schizophrenia (Gunduz-Bruce, 2009) and is known to induce schizophrenia-like behaviors in humans (Krystal et al., 2003). For the reasons above, ketamine is not widely used for the treatment of MDD.

One proposed solution involves ketamine treatment in addition to the mood stabilizer lithium, and preclinically it has been shown to potentiate the behavioral and molecular antidepressant-like effects of ketamine in rodent models of depression (Liu et al., 2013; Chiu et al., 2014). Lithium is known to directly inhibit both isoforms of GSK-3 (Freland and Beaulieu, 2012), and can also indirectly inactivate GSK-3 through Akt, which phosphorylates GSK-3's negative regulatory site (Chalecka-Franaszek and Chuang, 1999). Both the kinases Akt and p70S6K, which are involved in the mTOR pathway, can phosphorylate and and inhibit GSK-3 (Sutherland et al., 1993; Cross et al., 1995). In mouse kidney tubule cells, lithium treatment activated mTORC1 (Gao et al., 2013), however, less is known about lithium's interactions with mTORC2. Additionally, lithium can increase BDNF exon IV and promoter IV mRNA levels (Yasuda et al., 2009), suggesting that lithium may have a role in synaptogenesis (Zunszain et al., 2013). Indeed, adjunct lithium potentiated the frequency of serotonin- or hypocretin-induced excitatory postsynaptic currents in mPFC pyramidal cells, enhanced dendritic spine density and spine head diameter of mPFC layer V pyramidal neurons, increased mTOR and decreased GSK-3β signaling in the mPFC, and sustained the decreased FST immobility time of rats treated with a single low-dose ketamine injection (Liu et al., 2013). Long-term treatment of stressed mice with lithium in the drinking water potentiated and prolonged ketamine's molecular and behavioral antidepressant-like effects (Chiu et al., 2014). Specifically, post-ketamine lithium treatment demonstrated that lithium (1200 mg/L) prolonged antidepressant-like behavior and increased mPFC dendritic spine density of a single ketamine injection (50 mg/kg), up to 2 weeks. Pre-ketamine treatment with low-dose lithium (600 mg/L) for 2 weeks with a minimal dose of ketamine (2.5 mg/kg) acted synergistically to produce antidepressant-like behavior and molecular changes, at dosages that did not produce significant differences when administered separately.

Increased oxidative stress has been observed in psychiatric disorders such as MDD, schizophrenia and bipolar disorder, and has been suggested as a common underlying pathogenic mechanism (Ng et al., 2008). Significantly, increased plasma levels of malondialdehyde, a product of lipid peroxidation, glutathione peroxidase, an enzyme that reduces lipid hydroperoxides and free hydrogen peroxides, and other markers of oxidative stress were found in MDD patients compared to healthy controls (Sarandol et al., 2007). The increased levels of oxidative stress markers confer increased cellular susceptibility to oxidative damage from reactive oxygen species (ROS). While low-dose ketamine produces rapid and effective antidepressant results, one concern of ketamine treatment is its propensity to increase brain levels of harmful ROS. For instance, administration of sub-anesthetic doses of ketamine produced increased hydroxyl radical production and lipid peroxidation, and altered the activity of antioxidant enzymes, superoxide dismutase and catalase, in the rodent brain (Zuo et al., 2007; De Oliveira et al., 2009; Da Silva et al., 2010). In a chronic stress model, mice that also received a single ketamine injection (50 mg/kg) had significantly higher levels of lipid peroxidation, catalase activity, and oxidized glutathione, however, stressed animals that received lithium adjunct (1200 mg/L in drinking water) to ketamine treatment suppressed the high levels of oxidative stress in the PFC, hippocampus, and striatum (Chiu et al., 2014). Pre-ketamine lithium treatment neuroprotectively sustained lower levels of ROS, comparable with untreated stressed mice, highlighting the anti-oxidant properties of lithium.

Taken together, given the inevitable relapse of depressive symptoms and suicidal ideation, the preclinical results described above indicate that the GSK-3 inhibitor lithium, the mood stabilizer with strong anti-suicidal properties (Cipriani et al., 2005), may be a solution to this clinical issue, and provide a strong justification for the adjunctive use of lithium with ketamine in the treatment of depression. These results are consistent with a recent clinical observation that lithium-treated patients with bipolar disorder expressed greater anti-anhedonic responses to ketamine (Lally et al., 2014). In fact, several clinical trials are underway to assess the therapeutic benefits of lithium when given in conjunction with ketamine.

## Conclusion and future directions

Ketamine rapidly and robustly improves depressive symptoms and suicidal ideation of MDD subjects, and ameliorates behavioral and molecular deficits in animal models of depression. While preclinical experiments easily explore molecular targets of ketamine, the dosages used in preclinical studies have varied making it difficult to directly compare results from various studies. One explanation for the varying dosage requirements is that rodent strains respond to or metabolize ketamine differently. Thus, future preclinical studies should aim to understand why there are variations in ketamine dosage and response. Also notably, it has not been universally replicated that a single dose of ketamine produced sustained anti-depressant-like effects (Popik et al., 2008; Bechtholt-Gompf et al., 2011). The reason for this discrepancy deserves further investigations.

The working model shown in Figure 1 is to illustrate the possible mechanisms underlying the rapid antidepressant effects of ketamine and the potentiation of this efficacy by lithium. However, several issues need to be clarified. For example, given that ketamine seems to have similar affinity for pre- and post-synaptic NMDA receptors, what is the role of post-synaptic NMDA receptor inhibition in mediating ketamine's antidepressant effects? Lithium has been reported to be a blocker of NMDA receptors as shown by inhibition of receptor-mediated calcium influx in primary brain neurons (Hashimoto et al., 2002, 2003) and blockade of receptor-mediated activation of cytosolic phospholipase A2 in the rat brain (Rapoport, 2014). Could it be that the potentiation by lithium is contributed by further inhibition of NMDA receptor activity? Some of the pharmacologic effects of ketamine appear related to circulating metabolites of this drug (Zarate et al., 2012a). Future studies of the mechanisms of action of ketamine metabolites are also warranted.

Another issue with preclinical studies is the unequal numbers of male and female animals, resulting in less molecular and behavioral data regarding the female animal response to a sub-anesthetic dosage of ketamine. Many of the preclinical experiments only used male animals, but recent findings indicate that sex differences are present in the antidepressant effects of ketamine. For example, female Sprague-Dawley rats were more sensitive than males to the antidepressant effects of ketamine, responding to a lower dose (2.5 mg/kg), which was shown to be mediated by the gonadal hormones estrogen and progesterone (Carrier and Kabbaj, 2013). Therefore, future ketamine antidepressant research should include analysis of female animals, for enhanced research reproducibility, and for more accurate translation to clinical populations.

As an initial goal, we suggest that researchers study treatments, such as low-dose lithium, which can prolong ketamine's antidepressant effects greater than 1 week, or other agents which can cause rapid antidepressant actions with fewer side effects than ketamine, such as GLYX-13, an NMDA receptor glycine-site functional partial agonist (Burgdorf et al., 2013). However, more large-scale clinical and preclinical studies are needed to assess the safety and feasibility of ketamine and lithium adjunct for widespread clinical use. Lastly, it will be critical to determine all the specific synaptic mechanisms underlying ketamine's antidepressant actions to create better treatments for those suffering from depression.

### Conflict of interest statement

The authors declare that the research was conducted in the absence of any commercial or financial relationships that could be construed as a potential conflict of interest.

## Abbreviations

4E-BP1: 4E-binding protein 1
Akt/PKB: protein kinase B
AMPA: α-amino-3-hydroxy-5-methylisoxazole-4-propionic acid
Arc: activity-regulated cytoskeletal-associated protein
BDNF: brain-derived neurotrophic factor
eEF2K: eukaryotic elongation factor 2 kinase
eEF2: eukaryotic elongation factor 2
eIF4E: eukaryotic initiation factor 4E
ERK1/2: extracellular signal-regulated kinase
FST: forced swim test
GSK-3: glycogen synthase kinase-3
MDD: major depressive disorder
mPFC: medial prefrontal cortex
mTOR: mammalian target of rapamycin
NMDA: *N*-methyl-D-aspartate
p70S6K: p70 ribosomal protein S6 kinase
PFC: prefrontal cortex
PSD95: postsynaptic density protein 95
ROS: reactive oxygen species
TrkB: tropomyosin receptor kinase B.
